# Coexisting Asthma and Diabetes Are Associated With Adverse Metabolic and Inflammatory Profiles

**DOI:** 10.1111/dom.70767

**Published:** 2026-04-26

**Authors:** Sixtus Aguree, Patricia Silveyra, Nianjun Liu, Arthur H. Owora, Tesfaye B. Mersha

**Affiliations:** ^1^ Department of Applied Health Science, School of Public Health‐Bloomington Indiana University Bloomington Indiana USA; ^2^ Department of Environmental and Occupational Health, School of Public Health‐Bloomington Indiana University Bloomington Indiana USA; ^3^ Division of Pulmonary, Critical Care, Sleep, and Occupational Medicine, Department of Medicine, School of Medicine Indiana University Indianapolis Indiana USA; ^4^ Department of Epidemiology and Biostatistics, School of Public Health Indiana University Bloomington Indiana USA; ^5^ Department of Pediatrics, School of Medicine Indiana University Indianapolis Indiana USA; ^6^ Department of Medicine, School of Medicine Indiana University Indianapolis Indiana USA

**Keywords:** asthma, cardiometabolic risk, comorbidity, insulin resistance, interaction analysis, NHANES, systemic inflammation, type 2 diabetes, waist circumference

## Abstract

**Aims:**

Asthma and diabetes frequently co‐occur and share metabolic and inflammatory features, yet the cardiometabolic profile associated with their coexistence is not well defined. We examined differences in cardiometabolic and inflammatory biomarkers by joint asthma–diabetes status in U.S. adults and evaluated whether current asthma modifies diabetes‐associated alterations.

**Methods:**

We conducted a cross‐sectional analysis of 5295 non‐pregnant adults aged 20–85 years from NHANES 2015–2020 with complete fasting data. Participants were categorized as having neither condition (77.0%), asthma only (7.0%), diabetes only (14.1%), or both conditions (1.9%). Survey‐weighted multivariable linear regression estimated associations with glycaemic, lipid, insulin‐resistance and inflammatory biomarkers. Multiplicative and additive interactions were assessed using product terms and the relative excess risk due to interaction (RERI). Models were adjusted for sociodemographic and behavioural factors and waist circumference.

**Results:**

Participants with both asthma and diabetes displayed the most adverse biomarker profile. Compared with those with neither condition, the comorbid group had higher fasting glucose (*β* 58.86 mg/dL, 95% CI 45.49–72.23), HbA1c (*β* 1.70%, 95% CI 1.38–2.03), HOMA‐IR (*β* 5.93, 95% CI 3.43–8.42), TyG index (*β* 0.75, 95% CI 0.56–0.95) and ln(triglycerides) (*β* 0.35, 95% CI 0.14–0.56). The largest differences were observed for inflammatory markers, including ln(hs‐CRP) (*β* 0.74, 95% CI 0.35–1.12) and hs‐CRP (*β* 6.88 mg/L, 95% CI 2.08–11.67). Adjusted predicted means for fasting glucose (158.8 mg/dL, 95% CI 144.9–172.8) and HbA1c (7.07%, 95% CI 6.74–7.41) were also highest in the comorbid group. Multiplicative interaction tests indicated no interaction for glycaemic or lipid outcomes; significant interactions were limited to HDL‐C (*p* = 0.039) and SIRI (*p* = 0.003), both reflecting sub‐additive effects. No additive interaction remained significant after multiple‐testing correction.

**Conclusions:**

Coexisting asthma and diabetes are associated with a substantially greater burden of dysglycaemia, insulin resistance and inflammation than either condition alone. However, formal interaction analyses suggest that these joint effects are not synergistic and instead follow additive or sub‐additive patterns.

## 1 Introduction

According to the Global Initiative for Asthma and the International Diabetes Federation, asthma affects more than 260 million people worldwide, while diabetes is projected to impact approximately 589 million individuals by 2025. These chronic conditions place a significant economic burden globally, with asthma‐related costs exceeding US $100 billion annually and diabetes‐related healthcare expenditures surpassing US $1 trillion [1, 2]. The coexistence of asthma and diabetes defines a clinically significant population at potentially increased risk of cardiovascular complications, metabolic disorders and poor glycaemic control [3, 4]. Both asthma and diabetes are associated with chronic low‐grade inflammation and immune dysregulation. In asthma, airway inflammation is accompanied by systemic inflammatory signalling, including elevated circulating cytokines and acute‐phase reactants [5, 6, 7, 8]. In diabetes, particularly T2DM, metabolic dysfunction is closely linked to inflammatory processes involving adipose tissue, insulin resistance and altered immune responses [4, 9]. These shared features have led to growing interest in the interplay between respiratory and metabolic diseases, particularly in individuals with coexisting conditions.

Epidemiologic studies have reported associations between asthma and diabetes, including evidence suggesting bidirectional relationships in disease risk [9, 10]. However, most prior studies have focused on disease incidence or single biomarker domains and have often been limited by smaller sample sizes or selected populations. Moreover, relatively few studies have examined the broader cardiometabolic and inflammatory phenotype associated with asthma–diabetes comorbidity in nationally representative populations. An important limitation of existing literature is the reliance on descriptive comparisons without formal evaluation of interaction. Observed differences in biomarker levels among individuals with comorbid conditions have frequently been interpreted as evidence of synergistic effects, despite limited statistical assessment of whether joint effects exceed those expected from the individual conditions. Distinguishing between descriptive differences and formal interaction is essential for accurate interpretation of comorbidity‐related risk [11]. In addition, central adiposity represents a key shared pathway linking asthma and diabetes but has not been consistently accounted for in prior analyses; waist circumference may better capture cardiometabolic risk than body mass index (BMI), yet many studies have relied on BMI alone, potentially resulting in residual confounding [3, 4].

To address these gaps, we conducted a cross‐sectional analysis of nationally representative data from the National Health and Nutrition Examination Survey (NHANES) 2015–2020. We aimed to (1) characterize cardiometabolic and inflammatory biomarker profiles across four groups defined by joint asthma–diabetes status, and (2) formally assess whether current asthma modifies diabetes‐associated alterations in these biomarkers using both multiplicative and additive interaction frameworks, with rigorous control for confounding including central adiposity.

## 2 Materials and Methods

### 2.1 Study Design and Setting

This study is a cross‐sectional study using data from the NHANES, a nationally representative survey conducted by the National Center for Health Statistics (NCHS). NHANES uses a complex, multistage probability sampling strategy to capture a representative sample of the civilian, noninstitutionalized U.S. population. Data were pooled from survey cycles from 2015 through 2020 to increase statistical power and provide robust estimates of metabolic and inflammatory markers in individuals with asthma, diabetes, or both. All NHANES protocols were approved by the National Center for Health Statistics Research Ethics Review Board, and participants provided written informed consent. This secondary analysis of de‐identified publicly available data was conducted in accordance with STROBE guidelines.

### 2.2 Study Population

**FIGURE 1:**
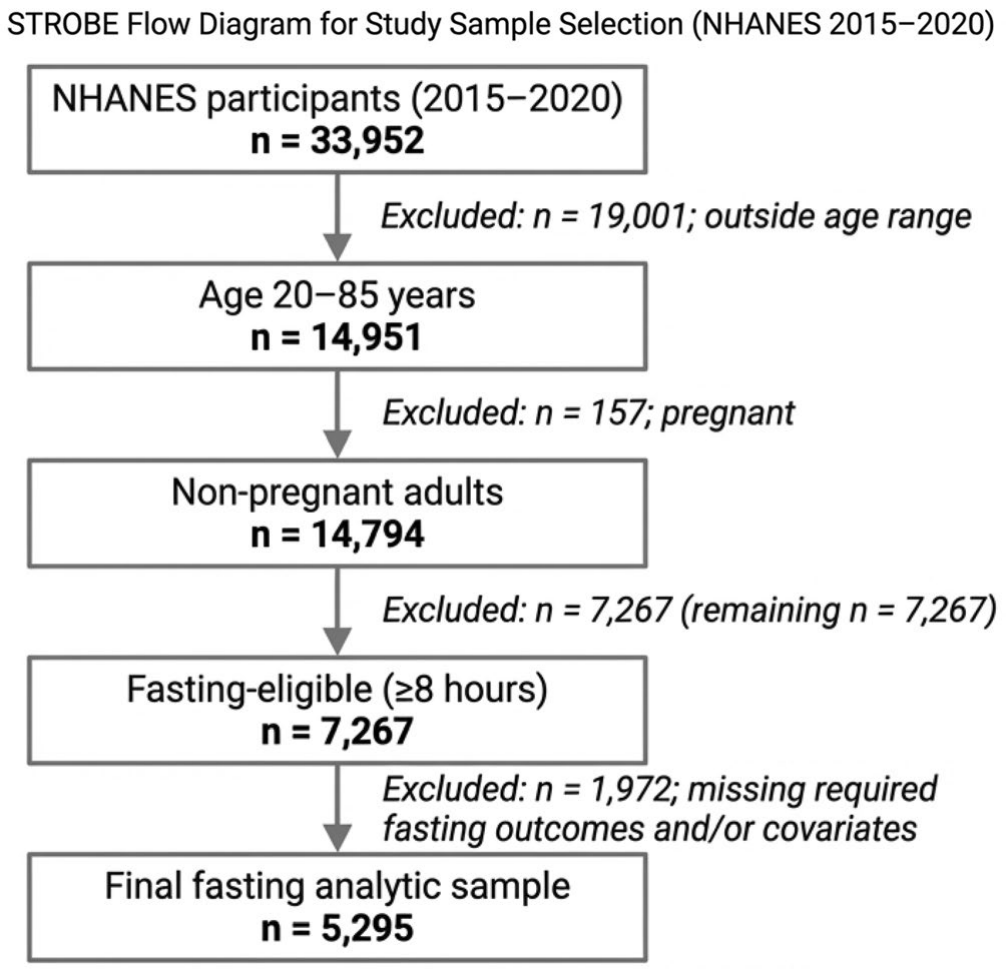
Study flow diagram of the NHANES 2015–2020 fasting analytic sample. Of 33 952 NHANES 2015–2020 participants, 19 001 were excluded for being outside the eligible age range of 20–85 years and 157 for pregnancy, leaving 14 794 age‐eligible non‐pregnant adults. Of these, 7267 met fasting eligibility criteria (≥ 8 h fasting with documented fasting time). The final fasting analytic sample comprised 5295 participants after additionally excluding 1972 individuals with missing data on required fasting laboratory measures (fasting plasma glucose, fasting insulin or triglycerides) or key model covariates. For SII and SIRI outcomes, the analytic sample was 5283 due to additional missing complete blood count components.

The initial NHANES 2015–2020 sample included 33 952 participants. Individuals were eligible if aged 20–85 years, not pregnant and met fasting requirements of at least 8 h prior to blood collection. After applying age, pregnancy and fasting exclusions, 7267 participants remained. Participants with missing data on cardiometabolic biomarkers or covariates were subsequently excluded, resulting in a final sample of 5295 individuals. Analyses involving the systemic immune‐inflammation index (SII) and systemic inflammation response index (SIRI) were conducted in a slightly smaller sample of 5283 participants due to exclusion of individuals with missing complete blood count data (Figure 1).

### 2.3 Exposure Classification

The primary exposure was joint asthma–diabetes status, categorized into four mutually exclusive groups: neither condition, asthma only, diabetes only and both conditions. Current asthma was defined by affirmative responses to both a prior physician diagnosis of asthma and still having asthma at the time of the survey [12]. Diabetes was defined as a self‐reported physician diagnosis or current use of insulin or oral glucose‐lowering medications [13]. This approach identifies diagnosed diabetes but does not distinguish between subtypes; however, in U.S. adults, approximately 90%–95% of diagnosed cases are T2DM [14]. This limitation is inherent to the data source and cannot be resolved analytically; biomarker‐confirmed subtyping would be required in future work.

### 2.4 Outcome Measures

Cardiometabolic and inflammatory biomarkers were assessed using standardized NHANES laboratory protocols [12]. Glycaemic measures included fasting plasma glucose (hexokinase method) and glycated haemoglobin (HbA1c; high‐performance liquid chromatography). Insulin resistance was estimated using HOMA‐IR = [fasting insulin (μU/mL) × fasting glucose (mg/dL)]/405 [15, 16]. A threshold of HOMA‐IR ≥ 2.5 was used to define moderate to high insulin resistance, consistent with established epidemiologic norms [17]. Serum total cholesterol and triglycerides were measured enzymatically using Roche/Hitachi Modular P or Cobas platforms. Participants were required to fast for at least 8 h for triglyceride assessment. Low‐density lipoprotein cholesterol (LDL‐C) was calculated using the Friedewald formula for participants with triglyceride levels < 400 mg/dL [18]. Systemic inflammatory indices were calculated using complete blood count parameters obtained from NHANES data [19].

High‐sensitivity CRP (hs‐CRP) was measured using a high‐sensitivity immunoturbidimetric assay on Roche Cobas c501 analysers. The Systemic Immune‐Inflammation Index (SII) was determined by multiplying the neutrophil count by the platelet count and then dividing that result by the lymphocyte count. The Systemic Inflammation Response Index (SIRI) was calculated by multiplying the neutrophil count by the monocyte count and then dividing that result by the lymphocyte count. Both indices were computed using absolute cell counts, expressed in thousands of cells per microliter (10^3^ cells/μL). SII = (neutrophil × platelet)/lymphocyte and SIRI = (neutrophil × monocyte)/lymphocyte were derived from complete blood count data [20, 21]. Blood pressure (BP) was measured by trained personnel following a standardized protocol. From 2015 to 2017, BP was assessed manually using a mercury sphygmomanometer and the auscultatory method, after participants had rested quietly in a seated position for 5 min. Up to three measurements were obtained, and the average of the available readings was used for analysis. During the 2017–2018 cycle, NHANES began transitioning to automated oscillometric BP measurements using the Omron HEM–907XL device. Both manual and automated methods were conducted in parallel during this cycle to assess comparability and calibration. Validation studies demonstrated strong agreement between the Omron and mercury devices, and from 2019 onward, the Omron HEM–907XL was adopted as the sole device for BP measurement in NHANES [22]. However, due to disruptions caused by the COVID‐19 pandemic, no BP data were collected during the 2019–2020 cycle. More details about NHANES data collection and method are available elsewhere [23, 24].

### 2.5 Covariates

Covariates were selected a priori based on clinical relevance and prior literature. Sociodemographic variables included age, sex, race/ethnicity, educational attainment and poverty–income ratio. Behavioural factors included smoking status (never: < 100 lifetime cigarettes; former: ≥ 100 lifetime cigarettes, not currently smoking; current: ≥ 100 lifetime cigarettes and currently smoking), alcohol use (never: < 12 lifetime drinks; former: ≥ 12 lifetime drinks, none in past 12 months; current: ≥ 12 lifetime drinks and ≥ 1 drink in past 12 months), physical activity (binary: meeting ≥ 150 min/week of moderate‐to‐vigorous activity [25]) and sleep duration (short: < 7 h/night; recommended: 7–9 h; long: > 9 h) [26]. Adiposity was assessed using continuous waist circumference as the primary measure, given its stronger association with cardiometabolic risk compared with BMI [27, 28].

### 2.6 Statistical Analysis

All analyses accounted for the complex NHANES survey design by incorporating sampling weights, clustering and stratification, with variance estimation using Taylor‐series linearization [29]. Descriptive statistics were presented as survey‐weighted means with standard errors for continuous variables and weighted proportions for categorical variables. Triglycerides and hs‐CRP were natural log‐transformed prior to regression due to right‐skewed distributions (absolute skewness > 1.0). Model assumptions were evaluated: linearity was assessed through residual plots with no meaningful departures identified; independence of observations is addressed structurally through the complex survey design specification, which incorporates clustering and stratification; and heteroscedasticity‐robust variance estimation via Taylor‐series linearization renders inference robust to violations of constant‐variance assumptions.

Associations between joint asthma–diabetes status and cardiometabolic outcomes were evaluated using survey‐weighted multivariable linear regression with four sequential model specifications: Model 1 (unadjusted); Model 2 (adjusted for age, sex, race/ethnicity, education and poverty–income ratio); Model 3 (Model 2 plus smoking status and continuous waist circumference); and Model 4 (Model 3 plus physical activity, alcohol use and sleep duration, representing the fully adjusted model). Effect modification by asthma was examined on both the multiplicative and additive scales. Multiplicative interaction was assessed by including product terms between the exposure variable and a binary indicator for asthma status (1 = asthma, 0 = no asthma) in the regression models and testing whether the coefficient for the product term differed from zero. Additive interaction was assessed by evaluating whether the combined effect of asthma and diabetes exceeded the sum of their individual effects. This was quantified using the relative excess risk due to interaction (RERI), the attributable proportion (AP) and the synergy index (S), which measure departures from additivity on the risk‐difference scale. The Benjamini–Hochberg false discovery rate procedure was applied to account for multiple comparisons in the interaction analyses [30]. The Benjamini–Hochberg false discovery rate procedure was applied to account for multiple comparisons in interaction analyses [31]. Pre‐specified subgroup analyses were conducted by sex and central obesity status. All analyses were performed in Stata version 19.5 (StataCorp, College Station, TX) [32].

### 2.7 Ethical Considerations

All NHANES protocols were approved by the National Center for Health Statistics Research Ethics Review Board, and all participants provided written informed consent. This study was conducted with publicly available, de‐identified data and was exempted from Institutional Review Board (IRB) approval.

## 3 Results

### 3.1 Study Population

The analytic sample included 5295 non‐pregnant adults aged 20–85 years. Overall, 77.0% had neither condition, 7.0% had asthma only, 14.1% had diabetes only and 1.9% had both asthma and diabetes. Participant characteristics by joint asthma–diabetes status are shown in Table 1. Individuals with both conditions were older (mean age 55.5 years) and predominantly female (72.5%). This group had the highest prevalence of obesity (78.8%) and central obesity (91.1%), the lowest proportion meeting physical activity guidelines (23.2%) and the highest prevalence of short sleep duration (33.9%). Persons with diabetes had a lower prevalence of higher educational attainment; this gradient should be interpreted in the context of cohort effects, as older adults with diabetes came of age in historical periods when educational attainment was substantially lower, rather than reflecting a direct causal pathway. Central adiposity was more prevalent across all disease groups relative to the reference group and is best characterized as a shared risk factor for both conditions rather than a consequence of either.

**TABLE 1:** Characteristics of the fasting analytic sample by joint asthma–diabetes status (NHANES 2015–2020).

Characteristic	Neither (*n* = 3789)	Asthma only (*n* = 347)	Diabetes only (*n* = 1007)	Both (*n* = 152)	*p*
Continuous variables, weighted mean ± SE					
Age, years	45.7 ± 0.5	46.9 ± 1.1	59.3 ± 0.6	55.5 ± 1.7	< 0.001
BMI, kg/m^2^	28.7 ± 0.2	30.1 ± 0.7	32.4 ± 0.4	37.6 ± 1.4	< 0.001
Waist circumference, cm	98.0 ± 0.5	101.5 ± 1.3	110.6 ± 0.8	119.8 ± 2.6	< 0.001
Fasting glucose, mg/dL	100.7 ± 0.3	100.2 ± 0.7	154.4 ± 2.6	161.4 ± 4.8	< 0.001
Fasting insulin, μU/mL	11.0 ± 0.2	12.7 ± 0.9	25.3 ± 2.0	25.3 ± 3.3	< 0.001
HbA1c, %	5.40 ± 0.01	5.43 ± 0.03	7.11 ± 0.07	7.31 ± 0.15	< 0.001
HOMA‐IR	2.81 ± 0.07	3.24 ± 0.26	9.95 ± 0.91	10.20 ± 1.05	< 0.001
TyG index	8.37 ± 0.01	8.42 ± 0.04	9.07 ± 0.04	9.20 ± 0.07	< 0.001
Triglycerides, mg/dL	100.8 ± 1.1	102.5 ± 3.8	135.7 ± 3.5	144.5 ± 7.5	< 0.001
ln(*Triglycerides*)	4.45 ± 0.01	4.51 ± 0.04	4.78 ± 0.03	4.87 ± 0.06	< 0.001
HDL‐C, mg/dL	56.1 ± 0.5	57.0 ± 1.3	48.0 ± 0.8	49.1 ± 1.2	< 0.001
LDL‐C, mg/dL	112.6 ± 0.7	113.5 ± 3.3	102.3 ± 2.2	104.8 ± 5.3	< 0.001
hs‐CRP, mg/L	3.3 ± 0.1	4.8 ± 0.8	5.0 ± 0.3	11.6 ± 1.8	< 0.001
ln(*hs‐CRP*)	0.46 ± 0.03	0.85 ± 0.12	0.96 ± 0.05	1.71 ± 0.17	< 0.001
SII	494.2 ± 8.2	545.6 ± 21.4	557.6 ± 15.3	638.4 ± 42.3	< 0.001
SIRI	1.14 ± 0.02	1.31 ± 0.06	1.48 ± 0.04	1.66 ± 0.15	< 0.001
Systolic BP, mmHg	120.5 ± 0.3	121.2 ± 0.9	130.1 ± 0.9	127.9 ± 2.1	< 0.001
Diastolic BP, mmHg	71.9 ± 0.3	72.8 ± 0.8	73.8 ± 0.6	75.0 ± 1.5	0.0027
Categorical variables, *n* (weighted column %)					
Age group					
20–39 years	1436 (41.1)	106 (37.8)	69 (8.7)	15 (13.4)	< 0.001
40–59 years	1270 (34.6)	135 (34.6)	337 (36.5)	55 (44.7)	
≥ 60 years	1083 (24.3)	106 (27.6)	601 (54.8)	82 (41.9)	
Sex					
Male	1876 (49.9)	114 (32.1)	562 (57.8)	47 (27.6)	< 0.001
Female	1913 (50.1)	233 (67.9)	445 (42.2)	105 (72.5)	
Race/ethnicity					
Non‐Hispanic White	1371 (64.4)	130 (64.2)	288 (58.7)	49 (61.2)	0.001
Non‐Hispanic Black	814 (10.4)	95 (13.2)	251 (13.1)	49 (16.7)	
Hispanic	955 (15.4)	71 (11.7)	316 (18.2)	39 (13.4)	
Non‐Hispanic Asian	481 (5.7)	22 (2.3)	109 (6.0)	4 (1.3)	
Other/multiracial	168 (4.1)	29 (8.5)	43 (4.0)	11 (7.4)	
BMI category					
Normal/underweight	1156 (30.5)	91 (28.3)	133 (11.1)	10 (5.3)	< 0.001
Overweight	1277 (34.4)	98 (26.1)	300 (28.6)	28 (15.9)	
Obesity	1332 (35.1)	155 (45.6)	563 (60.3)	114 (78.8)	
Waist circumference (central obesity)					
Normal	1751 (46.4)	117 (32.1)	224 (21.2)	14 (9.0)	< 0.001
High (central obesity)	1959 (53.6)	214 (67.9)	734 (78.8)	131 (91.1)	
Smoking status					
Never	2228 (57.1)	174 (51.9)	540 (52.0)	68 (38.9)	0.003
Former	842 (25.5)	90 (24.6)	309 (32.9)	51 (43.2)	
Current	716 (17.4)	82 (23.5)	156 (15.1)	33 (18.0)	
Physical activity (meets ≥ 150 min/week)					
No	2529 (63.3)	248 (69.5)	785 (73.9)	125 (76.8)	0.002
Yes	1258 (36.7)	98 (30.5)	218 (26.1)	27 (23.2)	
Sleep duration					
< 7 h	891 (20.6)	90 (25.9)	264 (24.5)	46 (33.9)	0.001
7–9 h	2514 (71.1)	200 (61.5)	599 (64.1)	81 (50.6)	
> 9 h	361 (8.3)	53 (12.6)	137 (11.4)	24 (15.5)	
Alcohol use					
Never	194 (7.0)	16 (4.5)	58 (11.5)	7 (4.5)	< 0.001
Former	381 (15.8)	44 (19.5)	174 (34.9)	33 (39.5)	
Current	1301 (77.1)	120 (76.0)	253 (53.6)	46 (56.0)	
Health insurance					
Yes	3087 (85.2)	301 (88.2)	880 (89.3)	135 (91.6)	0.040
No	694 (14.8)	44 (11.8)	127 (10.7)	16 (8.4)	
Family income‐to‐poverty ratio					
< 130% FPL	900 (18.5)	109 (21.6)	276 (21.4)	53 (29.7)	0.047
130%–349% FPL	1342 (34.6)	116 (40.8)	351 (38.3)	58 (40.5)	
≥ 350% FPL	1117 (46.9)	89 (37.6)	256 (40.3)	25 (29.8)	

*Note*: Values are survey‐weighted means ± SE (continuous) or unweighted *n* with weighted column % shown as *n* (%) (categorical). Analyses restricted to fasting subsample (fasting ≥ 8 h). *p*‐values from design‐based Wald tests (continuous) or Chi‐square tests (categorical). Log‐transformed rows (indented, italicized) presented on the natural‐log scale. SII = (Neutrophil × Platelet)/Lymphocyte; SIRI = (Neutrophil × Monocyte)/Lymphocyte (10^3^ cells/μL). Smoking definitions: never = never smoked ≥ 100 cigarettes; former = smoked ≥ 100 cigarettes but not currently; current = currently smokes. Physical activity: meets guideline if ≥ 150 min/week MVPA reported.

Abbreviations: BMI, body mass index; BP, blood pressure; HbA1c, glycated haemoglobin A1c; HDL‐C, high‐density lipoprotein cholesterol; HOMA‐IR, homeostatic model assessment of insulin resistance; hs‐CRP, high‐sensitivity C‐reactive protein; LDL‐C, low‐density lipoprotein cholesterol; SII, systemic immune‐inflammation index; SIRI, systemic inflammation response index; TyG, triglyceride–glucose index.

Cardiometabolic profiles differed across groups (all *p* < 0.001 unless otherwise noted). Participants with both asthma and diabetes had the highest mean BMI (37.6 kg/m^2^), waist circumference (119.8 cm), fasting glucose (161.4 mg/dL), HbA1c (7.31%), HOMA‐IR (10.20), TyG index (9.20), ln(triglycerides) (4.87) and ln(hs‐CRP) (1.71). SII (638.4) and SIRI (1.66) were also highest in this group. HDL‐C was lowest among participants with diabetes only (48.0 mg/dL). Characteristics by diabetes and asthma status separately (Tables S1 and S2) showed similar patterns.

### 3.2 Adjusted Associations

Fully adjusted estimates are shown in Table 2. Compared with participants with neither condition, diabetes‐only status was associated with higher fasting glucose (*β* 50.51 mg/dL, 95% CI 39.70–61.31), HbA1c (*β* 1.50%, 95% CI 1.29–1.71), fasting insulin (*β* 13.48 μU/mL, 95% CI 6.23–20.72), HOMA‐IR (*β* 6.28, 95% CI 3.89–8.67), TyG index (*β* 0.60, 95% CI 0.50–0.71) and ln(triglycerides) (*β* 0.25, 95% CI 0.18–0.33) and with lower HDL‐C (*β* −7.51 mg/dL, 95% CI −9.14 to −5.89) (all *p* < 0.001).

**TABLE 2:** Adjusted associations of joint asthma–diabetes status with cardiometabolic and inflammatory outcomes—fully adjusted Model 4 with continuous waist circumference (NHANES 2015–2020).

Outcome	Asthma only *β* (95% CI)	Diabetes only *β* (95% CI)	Both (asthma + diabetes) *β* (95% CI)	*p*‐int
Glycaemic and insulin‐resistance outcomes				
Fasting glucose, mg/dL	−0.04 (−2.26, 2.18)	50.51 (39.70, 61.31)***	58.86 (45.49, 72.23)***	0.235
Fasting insulin, μU/mL	0.02 (−2.04, 2.08)	13.48 (6.23, 20.72)***	9.90 (3.79, 16.02)**	0.122
HbA1c, %	−0.01 (−0.10, 0.09)	1.50 (1.29, 1.71)***	1.70 (1.38, 2.03)***	0.248
HOMA‐IR	−0.04 (−0.63, 0.54)	6.28 (3.89, 8.67)***	5.93 (3.43, 8.42)***	0.432
Lipid outcomes				
TyG index	0.11 (0.03, 0.19)**	0.60 (0.50, 0.71)***	0.75 (0.56, 0.95)***	0.611
ln(*Triglycerides*)	0.12 (0.05, 0.19)**	0.25 (0.18, 0.33)***	0.35 (0.14, 0.56)**	0.331
HDL‐C, mg/dL	−0.95 (−4.34, 2.43)	−7.51 (−9.14, −5.89)***	−5.84 (−10.11, −1.56)**	0.039*
LDL‐C, mg/dL	2.62 (−4.43, 9.67)	−14.57 (−21.90, −7.24)***	−13.67 (−32.24, 4.91)	0.851
Inflammatory outcomes				
ln(*hs‐CRP*)	0.29 (−0.10, 0.68)	0.26 (0.07, 0.45)**	0.74 (0.35, 1.12)***	0.649
SII	100.9 (24.6, 177.2)**	50.6 (−9.0, 110.2)	44.3 (−91.0, 179.6)	0.116
SIRI	0.34 (0.18, 0.50)***	0.22 (0.09, 0.35)**	0.10 (−0.15, 0.34)	0.003*
Blood pressure outcomes				
Systolic BP, mmHg	2.08 (0.24, 3.92)*	1.42 (−2.79, 5.62)	−0.13 (−6.41, 6.15)	0.307
Diastolic BP, mmHg	0.74 (−1.41, 2.89)	0.31 (−2.22, 2.85)	0.93 (−3.81, 5.67)	0.630

*Note*: Reference group: participants with neither asthma nor diabetes. *β* coefficients represent adjusted mean differences versus reference. *p*‐int: *p*‐value for the multiplicative diabetes × asthma interaction term. * Indicates nominally significant interaction (*p* < 0.05). Model 4B: adjusted for age, sex, race/ethnicity, education, poverty–income ratio, smoking status, continuous waist circumference, physical activity (≥ 150 min/week), alcohol use and sleep duration. Survey‐weighted linear regression with NHANES complex sampling design, fasting subpopulation. SII = (Neutrophil × Platelet)/Lymphocyte; SIRI = (Neutrophil × Monocyte)/Lymphocyte. Significance: *p < 0.05; **p < 0.01; ***p < 0.001.

Participants with both asthma and diabetes demonstrated higher fasting glucose (*β* 58.86 mg/dL, 95% CI 45.49–72.23; *p* < 0.001), HbA1c (*β* 1.70%, 95% CI 1.38–2.03; *p* < 0.001), HOMA‐IR (*β* 5.93, 95% CI 3.43–8.42; *p* < 0.001), TyG index (*β* 0.75, 95% CI 0.56–0.95; *p* < 0.001) and ln(triglycerides) (*β* 0.35, 95% CI 0.14–0.56; *p* < 0.01), compared with participants with neither condition. Inflammatory markers were also higher, ln(hs‐CRP) (*β* 0.74, 95% CI 0.35–1.12; *p* < 0.001). Asthma‐only status was not associated with glycaemic outcomes but was associated with higher TyG index (*β* 0.11, 95% CI 0.03–0.19; *p* < 0.01), ln(triglycerides) (*β* 0.12, 95% CI 0.05–0.19; *p* < 0.01), SII (*β* 100.9, 95% CI 24.6–177.2; *p* < 0.01), SIRI (*β* 0.34, 95% CI 0.18–0.50; *p* < 0.001) and systolic blood pressure (*β* 2.08 mmHg, 95% CI 0.24–3.92; *p* < 0.05). Sequential models (Table S3) showed attenuation after adjustment for waist circumference and behavioural factors.

### 3.3 Interaction Analyses

Multiplicative interaction analyses (Table 3), which tested whether the coefficient for the exposure × asthma product term differed from zero, showed no evidence of interaction between asthma and diabetes for glycaemic or insulin‐resistance outcomes, including fasting glucose (*p* = 0.235), HbA1c (*p* = 0.248) and HOMA‐IR (*p* = 0.432). No multiplicative interaction was observed for ln(triglycerides). Significant multiplicative interactions were detected for HDL‐C (interaction *β* 5.37, 95% CI 0.29–10.46; *p* = 0.039) and SIRI (interaction *β* −0.50, 95% CI −0.81 to −0.19; *p* = 0.003). Additive interaction analyses, which evaluated whether the combined effect of asthma and diabetes exceeded the sum of their individual effects, showed nominal departures from additivity for high HOMA‐IR (RERI −0.409; *p* = 0.007) and high fasting glucose (RERI 0.315; *p* = 0.036) (Table 4). However, neither remained significant after correction for multiple testing. No other additive interactions were observed. Sensitivity analyses (Table S4) demonstrated consistent findings across model specifications.

**TABLE 3:** Tests of multiplicative interaction between diabetes and current asthma on cardiometabolic and inflammatory outcomes (NHANES 2015–2020).

Outcome	Diabetes *β* (95% CI)	Current asthma *β* (95% CI)	Diabetes × asthma *β* (95% CI)	*p*‐int	Interpretation
Glycaemic & insulin‐resistance outcomes					
Fasting glucose, mg/dL	50.0 (38.9, 61.1)***	−0.17 (−2.34, 2.00)	7.66 (−5.29, 20.60)	0.235	No interaction
Fasting insulin, μU/mL	11.2 (4.8, 17.6)***	−0.50 (−2.28, 1.28)	−7.20 (−16.45, 2.06)	0.122	No interaction
HbA1c, %	1.48 (1.27, 1.70)***	−0.01 (−0.10, 0.08)	0.18 (−0.13, 0.49)	0.248	No interaction
HOMA‐IR	5.61 (3.47, 7.75)***	−0.20 (−0.72, 0.32)	−1.35 (−4.84, 2.13)	0.432	No interaction
Lipid outcomes					
TyG index	0.56 (0.45, 0.67)***	0.10 (0.02, 0.19)*	−0.05 (−0.23, 0.14)	0.611	No interaction
ln(*Triglycerides*)	0.21 (0.14, 0.29)***	0.11 (0.03, 0.19)**	−0.10 (−0.30, 0.10)	0.331	No interaction
HDL‐C, mg/dL	−6.08 (−7.63, −4.54)***	−0.67 (−3.74, 2.40)	5.37 (0.29, 10.46)*	0.039	Significant*
LDL‐C, mg/dL	−14.3 (−21.5, −7.2)***	2.75 (−4.26, 9.76)	−1.90 (−22.52, 18.72)	0.851	No interaction
Inflammatory markers					
ln(*hs‐CRP*)	0.08 (−0.13, 0.29)	0.25 (−0.11, 0.60)	−0.09 (−0.51, 0.32)	0.649	No interaction
Immune‐inflammatory indices					
SII	37.1 (−20.1, 94.3)	97.3 (20.9, 173.7)*	−124.5 (−282.3, 33.2)	0.116	No interaction
SIRI	0.20 (0.06, 0.33)**	0.33 (0.17, 0.50)***	−0.50 (−0.81, −0.19)**	0.003	Significant**
Blood pressure outcomes					
Systolic BP, mmHg	1.44 (−2.82, 5.71)	2.12 (0.30, 3.93)*	−3.90 (−11.60, 3.81)	0.307	No interaction
Diastolic BP, mmHg	−0.47 (−3.17, 2.22)	0.58 (−1.65, 2.81)	−1.53 (−7.98, 4.93)	0.630	No interaction

*Note*: Survey‐weighted linear regression including diabetes × current asthma product term, fasting subpopulation. Covariates: age, sex, race/ethnicity, education, poverty–income ratio, smoking, continuous waist circumference, physical activity, alcohol use, sleep duration. Significant multiplicative interactions: HDL‐C (*p* = 0.039) and SIRI (*p* = 0.003). Suggestive interaction for hs‐CRP (*p* = 0.083). The negative SIRI interaction coefficient indicates a sub‐additive response in the comorbid group. Log‐transformed rows (indented, italicized) on the natural‐log scale. Significance: *p < 0.05; **p < 0.01; ***p < 0.001.

**TABLE 4:** Additive interaction between diabetes and current asthma on binary cardiometabolic outcomes, with multiple‐testing correction (NHANES 2015–2020).

Binary outcome	DM only PR_10_ (95% CI)	Asthma only PR_01_ (95% CI)	Both PR_11_ (95% CI)	RERI (95% CI)	AP (95% CI)	S	*p*‐int (Q‐BH)
High HOMA‐IR (≥ 2.5)	1.46 (1.35, 1.57)	1.06 (0.88, 1.24)	1.11 (0.92, 1.30)	−0.409 (−0.666, −0.151)	−0.368 (−0.631, −0.105)	0.214	0.007 (0.066)
Metabolic syndrome (≥ 3 criteria)	1.49 (1.35, 1.62)	0.99 (0.80, 1.19)	1.19 (0.95, 1.42)	−0.294 (−0.602, 0.013)	−0.248 (−0.535, 0.039)	NE	0.121 (0.607)
High hs‐CRP (> 3 mg/L)	1.07 (0.94, 1.19)	1.11 (0.84, 1.38)	1.00 (0.76, 1.24)	−0.174 (−0.493, 0.144)	−0.174 (−0.510, 0.163)	0.024	0.280 (0.860)
High fasting glucose (≥ 100 mg/dL)	1.35 (1.26, 1.44)	0.93 (0.69, 1.16)	1.59 (1.40, 1.78)	0.315 (0.021, 0.609)	0.199 (0.025, 0.372)	2.159	0.036 (0.180)
High blood pressure (≥ 130/80 mmHg)	1.34 (1.21, 1.47)	1.20 (0.94, 1.45)	1.48 (1.33, 1.64)	−0.050 (−0.386, 0.285)	−0.034 (−0.261, 0.193)	0.906	0.769 (0.860)
Low HDL (sex‐specific cut‐offs)	1.52 (1.20, 1.83)	0.97 (0.69, 1.25)	1.27 (1.00, 1.54)	−0.219 (−0.691, 0.253)	−0.172 (−0.551, 0.207)	NE	0.465 (0.860)
High LDL (≥ 130 mg/dL)	0.58 (0.45, 0.71)	0.94 (0.69, 1.19)	0.69 (0.37, 1.00)	0.165 (−0.285, 0.615)	0.241 (−0.336, 0.817)	NE	0.469 (0.860)
High triglycerides (≥ 150 mg/dL)	1.78 (1.45, 2.11)	1.23 (0.91, 1.54)	2.34 (1.71, 2.98)	0.337 (−0.227, 0.902)	0.144 (−0.071, 0.359)	1.335	0.649 (0.860)
High TyG index (≥ 8.8)	1.94 (1.71, 2.17)	1.05 (0.78, 1.32)	2.15 (1.70, 2.61)	0.166 (−0.283, 0.614)	0.077 (−0.122, 0.276)	1.168	0.710 (0.860)
High SII (≥ 600)	1.06 (0.85, 1.27)	1.17 (0.91, 1.43)	1.11 (0.74, 1.48)	−0.118 (−0.540, 0.305)	−0.106 (−0.511, 0.299)	0.489	0.572 (0.860)
High SIRI (≥ 2)	1.32 (0.97, 1.67)	1.32 (0.71, 1.92)	1.92 (1.14, 2.70)	0.289 (−0.810, 1.388)	0.150 (−0.375, 0.676)	1.456	0.774 (0.860)

*Note*: PR_10_: prevalence ratio, diabetes‐only versus neither; PR_01_: asthma‐only versus neither; PR_11_: both versus neither. RERI and AP estimated from survey‐weighted Poisson regression with robust variance. S (synergy index) = point estimate only (delta‐method 95% CIs unstable in some strata). *p*‐int: raw interaction *p*‐value; Q‐BH: Benjamini–Hochberg adjusted *q*‐value. Nominal significance (*p* < 0.05) for High HOMA‐IR (RERI *p* = 0.007) and High fasting glucose (RERI *p* = 0.036); neither survived multiple‐testing correction (*Q* > 0.05). NE: synergy index not estimable when PR_11_ ≤ max(PR_10_, PR_01_). Outcome definitions: High fasting glucose ≥ 100 mg/dL; High HOMA‐IR ≥ 2.5; High triglycerides ≥ 150 mg/dL; High hs‐CRP > 3 mg/L; Low HDL < 40 mg/dL (men) or < 50 mg/dL (women); High BP ≥ 130/80 mmHg; High TyG ≥ 8.8; High SII ≥ 600; High SIRI ≥ 2. Models adjusted for age, sex, race/ethnicity, education, poverty–income ratio, smoking, physical activity, alcohol use, sleep duration and waist circumference. Other abbreviations as in Table 1.

Abbreviations: AP, attributable proportion; BH, Benjamini–Hochberg; DM, diabetes mellitus; NE, not estimable; PR, prevalence ratio; RERI, relative excess risk due to interaction; S, synergy index.

### 3.4 Adjusted Predicted Means

**FIGURE 2:**
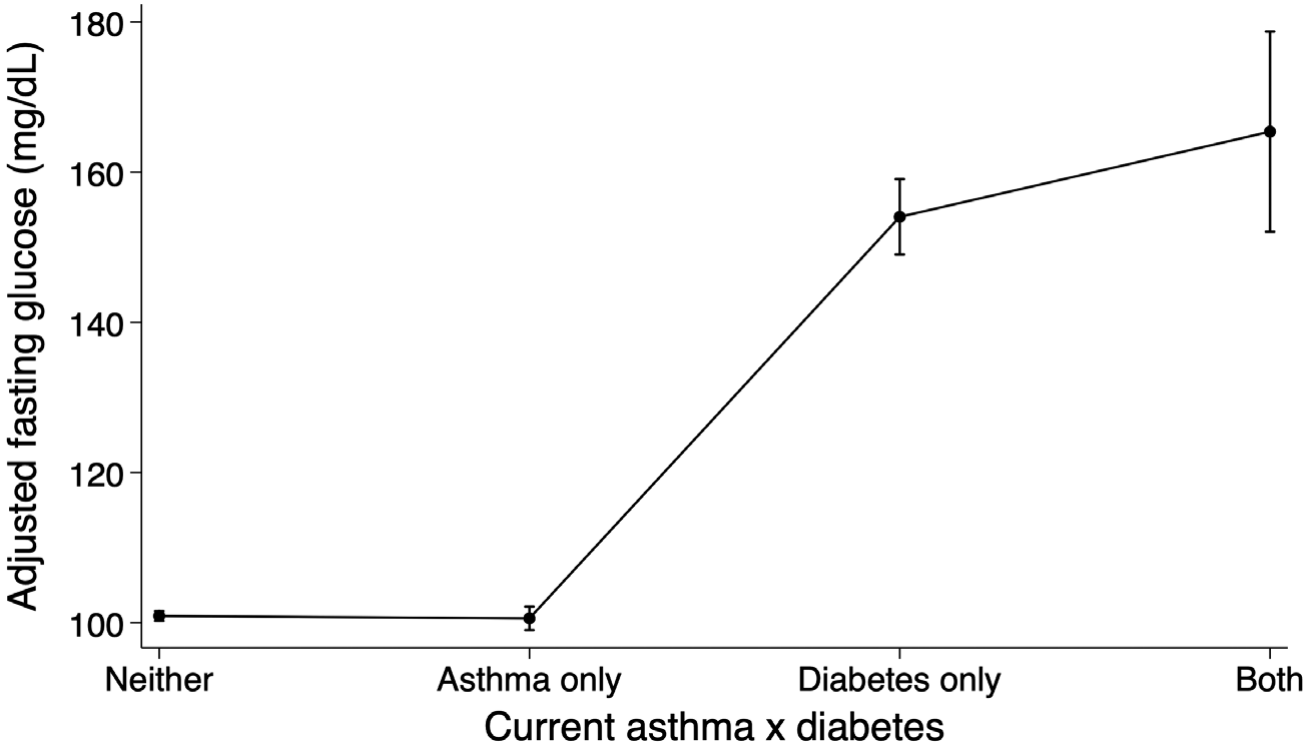
Adjusted predicted means (marginal effects) for fasting plasma glucose (mg/dL) by joint asthma–diabetes status, NHANES 2015–2020 fasting analytic sample (*N* = 5295). Points represent survey‐weighted adjusted predicted means; vertical bars denote 95% confidence intervals. The model was adjusted for age (continuous), sex, race/ethnicity, education, poverty–income ratio, smoking status, continuous waist circumference, physical activity (≥ 150 min/week guideline), alcohol use category and sleep duration category. Neither condition served as the reference group. Fasting glucose concentrations in the neither group (101.3 mg/dL, 95% CI: 100.3–102.4) and asthma‐only group (101.2 mg/dL, 95% CI: 99.3–103.0) were statistically indistinguishable and within the normoglycaemic range. Both the diabetes‐only group (151.3 mg/dL, 95% CI: 140.7–162.0) and the comorbid group (158.8 mg/dL, 95% CI: 144.9–172.8) had markedly elevated concentrations indicative of poor glycaemic control.

Participants with both asthma and diabetes had the highest predicted fasting glucose (158.8 mg/dL, 95% CI 144.9–172.8) and HbA1c (7.07%, 95% CI 6.74–7.41) (Table 5), followed by the diabetes‐only group (151.3 mg/dL; 6.91%) and the reference group (101.3 mg/dL; 5.43%) (Figure 2). Predicted values for the asthma‐only group were similar to the reference group. Subgroup analyses (Table S5) showed no evidence of effect modification.

**TABLE 5:** Adjusted predicted means (marginal effects) by joint diabetes–asthma status for cardiometabolic and inflammatory outcomes (NHANES 2015–2020).

Outcome	Neither mean (SE) [95% CI]	Asthma only mean (SE) [95% CI]	Diabetes only mean (SE) [95% CI]	Both (DM + asthma) mean (SE) [95% CI]
Glycaemic & insulin‐resistance outcomes				
Fasting glucose, mg/dL	101.3 [100.3, 102.4]^cd^	101.2 [99.3, 103.0]^cd^	151.3 [140.7, 162.0]^ab^	158.8 [144.9, 172.8]^ab^
Fasting insulin, μU/mL	12.0 [11.3, 12.6]^cd^	11.5 [9.6, 13.4]^c^	23.2 [16.7, 29.7]^ab^	15.5 [9.3, 21.7]^a^
HbA1c, %	5.43 [5.40, 5.45]^cd^	5.41 [5.32, 5.50]^cd^	6.91 [6.69, 7.13]^ab^	7.07 [6.74, 7.41]^ab^
HOMA‐IR	3.10 [2.93, 3.27]^cd^	2.89 [2.35, 3.44]^cd^	8.70 [6.54, 10.86]^ab^	7.15 [4.73, 9.57]^ab^

*Note*: Adjusted predicted means estimated from survey‐weighted linear regression using predictive margins (Stata margins command). Values presented as mean (SE) [95% CI]. Model 4B adjusted for age, sex, race/ethnicity, education, poverty–income ratio, smoking status, continuous waist circumference, physical activity, alcohol use and sleep duration. Superscript notation: ^a = Neither, ^b = Asthma Only, ^c = Diabetes Only, ^d = Both. Superscripts indicate significant difference (*p* < 0.05) from that group based on non‐overlapping 95% CIs. Total *N* = 4119 (fasting *N* = 2234) for all outcomes except SII/SIRI (*N* = 4117; fasting *N* = 2232).

## 4 Discussion

In this nationally representative cross‐sectional analysis of U.S. adults, asthma–diabetes comorbidity was associated with a distinct and clinically adverse cardiometabolic and inflammatory profile. Diabetes was the primary driver of metabolic dysfunction across all domains examined, whereas asthma alone was characterized predominantly by elevated inflammatory markers and central adiposity, without independent associations with glycaemic outcomes after full adjustment of other factors. Individuals with both conditions consistently exhibited the highest absolute levels of fasting glucose, HbA1c and systemic inflammation compared with those with either condition alone.

Importantly, formal interaction analyses demonstrated that these joint effects are not synergistically amplified. Significant multiplicative interactions were observed for HDL cholesterol and SIRI, both in a negative direction consistent with sub‐additive effects, while no statistically robust additive interaction was detected after correction for multiple comparisons. These findings indicate that the excess burden observed in the comorbid group reflects higher absolute risk rather than biological amplification beyond the expected combined effects of asthma and diabetes, a distinction of considerable importance for both causal inference and clinical communication.

These findings are consistent with prior studies reporting associations between asthma and diabetes and extend this literature by providing a comprehensive evaluation of cardiometabolic and inflammatory biomarkers in a nationally representative population [33, 34]. Previous work has shown that diabetes is associated with adverse metabolic and inflammatory profiles, while asthma has been linked more selectively to systemic inflammation and adiposity [35, 36]. Most prior studies, however, have relied on descriptive comparisons without formally evaluating interaction, and higher biomarker levels in comorbid populations have often been interpreted as evidence of synergistic effects. By formally testing interaction, our analysis shows that although individuals with both conditions have worse absolute biomarker profiles, the combined effects are largely consistent with additive expectations after adjustment for confounders.

A key finding is the central role of waist circumference in shaping the observed associations. Adjustment for continuous waist circumference attenuated several cardiometabolic associations, particularly for glycaemic outcomes, highlighting central adiposity as a major shared pathway linking asthma and diabetes. Notably, multiplicative interaction effects for glycaemic outcomes that were nominally significant in models using BMI category became non‐significant after substituting continuous waist circumference, underscoring the importance of adiposity measurement choice in comorbidity research [37, 38]. These results suggest that a substantial portion of the excess burden in individuals with both conditions is attributable to shared adiposity‐related mechanisms rather than disease‐specific biological interaction.

There are several important clinical implications. Adults with coexisting asthma and diabetes demonstrate consistently higher levels of hyperglycaemia and systemic inflammation, both of which are associated with an increased risk of cardiovascular and microvascular complications. In this cohort, the mean HbA1c (comorbid group) of 7.07% exceeded the standard threshold for poor glycaemic control, while the geometric mean hs‐CRP of 5.53 mg/L placed patients within a high cardiovascular risk category [39]. These findings support intensified glycaemic monitoring in this population, including consideration of continuous glucose monitoring (CGM), particularly during periods of systemic corticosteroid use when postprandial hyperglycaemia may be under‐recognized by HbA1c alone.

In this context, the metabolic consequences of systemic corticosteroid exposure warrant careful consideration, given their well‐established effects on hyperglycaemia and insulin resistance [40, 41, 42]. These risks are amplified in individuals with coexisting obesity or type 2 diabetes, underscoring the importance of minimizing cumulative oral corticosteroid (OCS) exposure in routine care [43, 44]. Biologic therapies targeting type 2 inflammation therefore represent an important steroid‐sparing strategy. Agents directed against IL‐5 and IL‐5 receptor (mepolizumab, benralizumab) and IL‐4 receptor α (dupilumab) have demonstrated consistent reductions in maintenance OCS use across clinical trials and real‐world studies [45, 46, 47, 48, 49]. Other biologics, including reslizumab and tezepelumab, may reduce exacerbation burden, although evidence for sustained OCS‐sparing effects remains less robust [50, 51, 52]. Collectively, these findings support an integrated, multidisciplinary approach that addresses airway inflammation alongside glycaemic control and obesity, particularly in patients with overlapping metabolic disease [53, 54].

Although this study was not designed to assess biological mechanisms directly, the observed patterns are consistent with shared pathways linking inflammation and metabolic dysfunction. Visceral adipose tissue may act as a common upstream driver through its role in producing proinflammatory cytokines, adipokines and acute‐phase reactants that affect both airway and metabolic physiology [55, 56]. The sub‐additive SIRI interaction may partly reflect the offsetting effects of corticosteroid therapy on leucocyte dynamics, producing neutrophilia and monocytosis while simultaneously suppressing lymphocyte counts [57, 58] and the partial counteraction between the Th2‐predominant immune environment of atopic asthma and the Th1‐predominant and innate immune activation characteristic of T2DM [59, 60]. These mechanistic interpretations remain hypothesis‐generating in the context of this cross‐sectional study.

Several limitations should be acknowledged. The cross‐sectional design precludes causal inference, and the observed associations may reflect pre‐existing susceptibility differences, reverse causality, or shared upstream determinants rather than consequences of comorbidity. Asthma and diabetes status were based on self‐report and medication use, which may introduce non‐differential misclassification that would likely attenuate associations toward the null. The diabetes definition does not distinguish between subtypes; however, given that T2DM accounts for approximately 90%–95% of diagnosed diabetes in U.S. adults, the findings predominantly reflect T2DM‐related processes. Residual confounding from unmeasured factors including diet quality, corticosteroid dose and duration and disease duration, cannot be excluded, and exclusion of participants with missing data may introduce selection bias. Finally, the small, unweighted sample of individuals with both conditions limited statistical power for interaction analyses.

This study also has important strengths. The use of a large, nationally representative dataset with comprehensive directly measured cardiometabolic and inflammatory biomarkers enables generalizable conclusions. Rigorous evaluation of interaction on both multiplicative and additive scales, with Benjamini–Hochberg correction for multiple comparisons, provides a statistically defensible framework for characterizing joint exposure effects—one that avoids the conflation of descriptive group differences with formal statistical interaction that has characterized much prior literature.

In conclusion, asthma–diabetes comorbidity is associated with a clinically distinct cardiometabolic and inflammatory profile characterized by higher absolute levels of dysglycaemia and systemic inflammation, but formal interaction analyses confirm that these joint effects are not synergistically amplified beyond what is expected from the individual conditions. Central adiposity is a critical shared confounding pathway. These findings support integrated risk assessment and management strategies in individuals with multimorbidity, including vigilance for dysglycaemia in asthma care and coordinated approaches targeting both airway disease and metabolic risk. Future prospective studies incorporating biomarker‐confirmed diabetes subtyping, corticosteroid burden quantification and mechanistic cytokine profiling are needed to establish the causal architecture of this clinically important comorbidity.

## Funding

The authors have nothing to report.

## Conflicts of Interest

The authors declare no conflicts of interest.

## Supporting information


**Table S1:** Characteristics of the fasting analytic sample by diabetes status (NHANES 2015–2020).
**Table S2:** Characteristics of the fasting analytic sample by current asthma status (NHANES 2015–2020).
**Table S3:** Sequential model results for joint asthma–diabetes status (Asthma + Diabetes) versus neither (NHANES 2015–2020).
**Table S4:** Sensitivity analyses: associations of joint asthma–diabetes status across alternative model specifications (NHANES 2015–2020).
**Table S5:** Subgroup analyses by sex and central obesity status—joint asthma–diabetes status (NHANES 2015–2020).

## Data Availability

This study used publicly available data from the U.S. National Health and Nutrition Examination Survey (NHANES), which can be accessed at https://www.cdc.gov/nchs/nhanes/. All data used in the analysis are anonymized and available to the public.
