# Supplementary material for: Coexisting Asthma and Diabetes Are Associated With Adverse Metabolic and Inflammatory Profiles
Source: Diabetes Obes Metab. 2026 Apr 26;28(7):6073–85. doi: 10.1111/dom.70767 (PMC13243984; doi:10.1111/dom.70767)
Supplement: Supplementary file 1 — Table S1: Characteristics of the fasting analytic sample by diabetes status (NHANES 2015–2020). Table S2: Characteristics of the fasting analytic sample by current asthma status (NHANES 2015–2020). Table S3: Sequential model results for joint asthma–diabetes status (Asthma + Diabetes) versus neither (NHANES 2015–2020). Table S4: Sensitivity analyses: associations of joint asthma–diabetes status across alternative model specifications (NHANES 2015–2020). Table S5: Subgroup analyses by sex and central obesity status—joint asthma–diabetes status (NHANES 2015–2020). [file DOM-28-6073-s001.docx]

**APPENDIX TABLES**

**Appendix Table S1. Characteristics of the fasting analytic sample by diabetes status (NHANES 2015–2020)**

| Characteristic | No Diabetes (n=4,136) | Diabetes (n=1,159) | Total (n=5,295) | P-value |
| --- | --- | --- | --- | --- |
| Continuous variables , weighted mean ± SE | | | | |
| Age, years | 45.8 ± 0.5 | 58.8 ± 0.6 | 47.9 ± 0.5 | <0.001 |
| BMI, kg/m² | 28.8 ± 0.2 | 33.0 ± 0.4 | 29.5 ± 0.2 | <0.001 |
| Waist circumference, cm | 98.3 ± 0.5 | 111.6 ± 0.8 | 100.4 ± 0.4 | <0.001 |
| Fasting glucose, mg/dL | 100.7 ± 0.3 | 155.2 ± 2.4 | 109.3 ± 0.6 | <0.001 |
| HbA1c, % | 5.40 ± 0.01 | 7.13 ± 0.07 | 5.67 ± 0.02 | <0.001 |
| HOMA-IR | 2.85 ± 0.07 | 9.98 ± 0.79 | 3.97 ± 0.13 | <0.001 |
| TyG index | 8.37 ± 0.01 | 9.09 ± 0.03 | 8.48 ± 0.01 | <0.001 |
| Triglycerides, mg/dL | 100.9 ± 1.1 | 136.7 ± 3.5 | 106.5 ± 1.3 | <0.001 |
| *ln(Triglycerides)* | 4.46 ± 0.01 | 4.79 ± 0.03 | 4.51 ± 0.01 | <0.001 |
| HDL-C, mg/dL | 56.1 ± 0.5 | 48.1 ± 0.8 | 54.9 ± 0.4 | <0.001 |
| LDL-C, mg/dL | 112.7 ± 0.7 | 102.6 ± 2.1 | 111.1 ± 0.8 | <0.001 |
| hs-CRP, mg/L | 3.4 ± 0.1 | 5.8 ± 0.3 | 3.8 ± 0.1 | <0.001 |
| *ln(hs-CRP)* | 0.49 ± 0.04 | 1.04 ± 0.05 | 0.57 ± 0.03 | <0.001 |
| SII | 498.4 ± 7.4 | 566.9 ± 14.7 | 509.1 ± 7.6 | <0.001 |
| SIRI | 1.15 ± 0.02 | 1.50 ± 0.05 | 1.21 ± 0.02 | <0.001 |
| Systolic BP, mmHg | 120.5 ± 0.3 | 129.8 ± 0.8 | 122.0 ± 0.3 | <0.001 |
| Diastolic BP, mmHg | 72.0 ± 0.3 | 74.0 ± 0.5 | 72.3 ± 0.3 | 0.001 |
| Categorical variables , n (weighted column %) | | | | |
| *Age group* | | | | |
| 20–39 years | 1,542 (40.8) | 84 (9.3) | 1,626 (35.8) | <0.001 |
| 40–59 years | 1,405 (34.6) | 392 (37.4) | 1,797 (35.0) |  |
| ≥60 years | 1,189 (24.6) | 683 (53.3) | 1,872 (29.2) |  |
| *Sex* | | | | |
| Male | 1,990 (48.4) | 609 (54.2) | 2,599 (49.3) | 0.033 |
| Female | 2,146 (51.6) | 550 (45.8) | 2,696 (50.7) |  |
| *Race/ethnicity* | | | | |
| Non-Hispanic White | 1,501 (64.4) | 337 (59.0) | 1,838 (63.5) | 0.039 |
| Non-Hispanic Black | 909 (10.6) | 300 (13.5) | 1,209 (11.1) |  |
| Hispanic | 1,026 (15.1) | 355 (17.6) | 1,381 (15.5) |  |
| Non-Hispanic Asian | 503 (5.5) | 113 (5.4) | 616 (5.5) |  |
| Other/multiracial | 197 (4.5) | 54 (4.4) | 251 (4.5) |  |
| *Education level* | | | | |
| <High school | 748 (11.1) | 330 (19.0) | 1,078 (12.4) | <0.001 |
| High school/GED | 952 (24.6) | 277 (27.4) | 1,229 (25.1) |  |
| >High school | 2,436 (64.2) | 552 (53.5) | 2,988 (62.5) |  |
| *BMI category* | | | | |
| Normal/underweight | 1,247 (30.3) | 143 (10.4) | 1,390 (27.2) | <0.001 |
| Overweight | 1,375 (33.7) | 328 (27.0) | 1,703 (32.6) |  |
| Obesity | 1,487 (36.0) | 677 (62.5) | 2,164 (40.2) |  |
| *Waist circumference (central obesity)* | | | | |
| Normal | 1,868 (45.2) | 238 (19.8) | 2,106 (41.2) | <0.001 |
| High (central obesity) | 2,268 (54.8) | 921 (80.2) | 3,189 (58.8) |  |
| *Smoking status* | | | | |
| Never | 2,402 (56.7) | 608 (50.4) | 3,010 (55.7) | 0.003 |
| Former | 932 (25.4) | 360 (34.1) | 1,292 (26.8) |  |
| Current | 798 (17.9) | 189 (15.5) | 987 (17.5) |  |
| *Physical activity (meets ≥150 min/wk)* | | | | |
| No | 2,777 (63.8) | 910 (74.2) | 3,687 (65.5) | 0.002 |
| Yes | 1,356 (36.2) | 245 (25.8) | 1,601 (34.5) |  |
| *Sleep duration* | | | | |
| <7 hours | 981 (21.1) | 310 (25.7) | 1,291 (21.8) | 0.003 |
| 7–9 hours | 2,714 (70.3) | 680 (62.4) | 3,394 (69.0) |  |
| >9 hours | 414 (8.6) | 161 (11.9) | 575 (9.1) |  |
| *Alcohol use* | | | | |
| Never drank | 210 (6.8) | 65 (10.6) | 275 (7.4) | <0.001 |
| Former drinker | 425 (16.1) | 207 (35.5) | 632 (19.2) |  |
| Current drinker | 1,421 (77.0) | 299 (53.9) | 1,720 (73.4) |  |
| *Current asthma* | | | | |
| No | 3,789 (91.7) | 1,007 (88.0) | 4,796 (91.1) | 0.002 |
| Yes | 347 (8.3) | 152 (12.0) | 499 (8.9) |  |

*Values are survey-weighted means ± SE (continuous) or unweighted n with weighted column % shown as n (%) (categorical). The education–diabetes association should be interpreted in the context of the strong age–education cohort effect. Log-transformed rows on the natural-log scale. Abbreviations: same as Table 1.*

**Appendix Table S2. Characteristics of the fasting analytic sample by current asthma status (NHANES 2015–2020)**

| Characteristic | No Current Asthma (n=4,796) | Current Asthma (n=499) | Total (n=5,295) | P-value |
| --- | --- | --- | --- | --- |
| Continuous variables , weighted mean ± SE | | | | |
| Age, years | 47.8 ± 0.5 | 48.7 ± 1.1 | 47.9 ± 0.5 | 0.370 |
| BMI, kg/m² | 29.3 ± 0.2 | 31.7 ± 0.6 | 29.5 ± 0.2 | <0.001 |
| Waist circumference, cm | 99.9 ± 0.4 | 105.3 ± 1.2 | 100.4 ± 0.4 | <0.001 |
| Fasting glucose, mg/dL | 108.9 ± 0.6 | 113.1 ± 2.0 | 109.3 ± 0.6 | 0.032 |
| HbA1c, % | 5.66 ± 0.02 | 5.83 ± 0.07 | 5.67 ± 0.02 | 0.010 |
| HOMA-IR | 3.90 ± 0.14 | 4.70 ± 0.37 | 3.97 ± 0.13 | 0.021 |
| TyG index | 8.48 ± 0.01 | 8.58 ± 0.04 | 8.48 ± 0.01 | 0.020 |
| ln(Triglycerides) | 4.50 ± 0.01 | 4.58 ± 0.03 | 4.51 ± 0.01 | 0.034 |
| HDL-C, mg/dL | 54.8 ± 0.4 | 55.3 ± 1.0 | 54.9 ± 0.4 | 0.894 |
| ln(hs-CRP) | 0.53 ± 0.03 | 1.03 ± 0.10 | 0.57 ± 0.03 | <0.001 |
| SII | 503.8 ± 8.0 | 565.1 ± 20.6 | 509.1 ± 7.6 | 0.006 |
| SIRI | 1.19 ± 0.02 | 1.38 ± 0.06 | 1.21 ± 0.02 | 0.001 |
| Systolic BP, mmHg | 121.9 ± 0.3 | 122.6 ± 0.9 | 122.0 ± 0.3 | 0.348 |
| Diastolic BP, mmHg | 72.2 ± 0.3 | 73.2 ± 0.7 | 72.3 ± 0.3 | 0.100 |
| Categorical variables , n (weighted column %) | | | | |
| Sex | | | | |
| Male | 2,438 (51.1) | 161 (31.1) | 2,599 (49.3) | <0.001 |
| Female | 2,358 (48.9) | 338 (68.9) | 2,696 (50.7) |  |
| Race/ethnicity | | | | |
| Non-Hispanic White | 1,659 (63.5) | 179 (63.5) | 1,838 (63.5) | 0.002 |
| Non-Hispanic Black | 1,065 (10.8) | 144 (14.0) | 1,209 (11.1) |  |
| Hispanic | 1,271 (15.8) | 110 (12.1) | 1,381 (15.5) |  |
| Non-Hispanic Asian | 590 (5.8) | 26 (2.1) | 616 (5.5) |  |
| Other/multiracial | 211 (4.1) | 40 (8.3) | 251 (4.5) |  |
| BMI category | | | | |
| Normal/underweight | 1,289 (27.5) | 101 (23.2) | 1,390 (27.2) | 0.004 |
| Overweight | 1,577 (33.5) | 126 (23.9) | 1,703 (32.6) |  |
| Obesity | 1,895 (39.0) | 269 (52.9) | 2,164 (40.2) |  |
| Waist circumference (central obesity) | | | | |
| Normal | 1,975 (42.5) | 131 (27.2) | 2,106 (41.2) | <0.001 |
| High (central obesity) | 2,693 (57.5) | 345 (72.8) | 3,038 (58.8) |  |
| Smoking status | | | | |
| Never | 2,768 (56.3) | 242 (49.1) | 3,010 (55.7) | 0.091 |
| Former | 1,151 (26.7) | 141 (28.6) | 1,292 (26.8) |  |
| Current | 872 (17.0) | 115 (22.3) | 987 (17.5) |  |
| Sleep duration | | | | |
| <7 hours | 1,155 (21.2) | 136 (27.7) | 1,291 (21.8) | 0.001 |
| 7–9 hours | 3,113 (70.0) | 281 (59.1) | 3,394 (69.1) |  |
| >9 hours | 498 (8.8) | 77 (13.2) | 575 (9.1) |  |
| Abdominal obesity | | | | |
| No | 1,975 (41.7) | 131 (25.8) | 2,106 (40.3) | <0.001 |
| Yes | 2,821 (58.3) | 368 (74.2) | 3,189 (59.7) |  |
| hs-CRP category | | | | |
| <1 mg/L | 1,480 (33.0) | 88 (21.8) | 1,568 (32.0) | <0.001 |
| 1–3 mg/L | 1,629 (34.9) | 153 (28.6) | 1,782 (34.3) |  |
| >3 mg/L | 1,687 (32.1) | 258 (49.6) | 1,945 (33.7) |  |
| Diabetes status | | | | |
| No | 3,789 (84.5) | 347 (78.4) | 4,136 (84.0) | 0.002 |
| Yes | 1,007 (15.5) | 152 (21.7) | 1,159 (16.0) |  |

*Values are survey-weighted means ± SE (continuous) or unweighted n with weighted column % shown as n (%) (categorical). Log-transformed rows on the natural-log scale. Abbreviations: same as Table 1.*

**Appendix Table S3. Sequential model results for joint asthma–diabetes status (Asthma + Diabetes) vs. Neither (NHANES 2015–2020)**

| Outcome | Model 1 (Unadjusted) | Model 2 (Demographic) | Model 3A (+ Smoking, Waist cat) | Model 3B (+ Smoking, Cont. Waist) | Model 4B (Fully Adjusted) | |
| --- | --- | --- | --- | --- | --- | --- |
| Both (Asthma + Diabetes) vs. Neither, β (95% CI) | | | | | | |
| Glycemic Outcomes | | | | | | |
| Fasting glucose, mg/dL | 65.8 (51.3, 80.2)*** | 69.7 (56.5, 83.0)*** | 63.6 (52.0, 75.3)*** | 61.8 (50.1, 73.4)*** | 58.9 (45.5, 72.2)*** | |
| HbA1c, % | 2.01 (1.61, 2.41)*** | 2.00 (1.61, 2.39)*** | 1.85 (1.52, 2.19)*** | 1.80 (1.47, 2.12)*** | 1.70 (1.38, 2.03)*** | |
| HOMA-IR | 8.17 (5.93, 10.42)*** | 7.77 (5.13, 10.41)*** | 6.17 (3.69, 8.64)*** | 4.52 (2.19, 6.84)*** | 5.93 (3.43, 8.42)*** | |
| TyG index | 0.90 (0.65, 1.14)*** | 0.94 (0.72, 1.16)*** | 0.79 (0.65, 0.92)*** | 0.64 (0.52, 0.77)*** | 0.75 (0.56, 0.95)*** | |
| Lipid Outcomes | | | | | | |
| Triglycerides, mg/dL | 52.0 (24.4, 79.7)*** | 57.4 (30.9, 83.9)*** | 41.2 (25.8, 56.5)*** | 29.8 (14.5, 45.1)*** | 39.1 (13.7, 64.5)*** | |
| HDL-C, mg/dL | −8.0 (−10.8, −5.2)*** | −11.4 (−14.2, −8.6)*** | −8.2 (−11.2, −5.2)*** | −3.7 (−6.2, −1.2)** | −5.8 (−10.1, −1.6)** | |
| LDL-C, mg/dL | −10.5 (−21.6, 0.5) | −11.1 (−24.9, 2.7) | −8.6 (−20.7, 3.5) | −10.8 (−23.6, 2.0) | −13.7 (−32.2, 4.9) | |
| Inflammatory Outcomes | | | | | | |
| ln(hs-CRP) | 1.29 (0.93, 1.65)*** | 1.19 (0.87, 1.52)*** | 0.89 (0.58, 1.20)*** | 0.37 (0.04, 0.70)* | 0.74 (0.35, 1.12)*** | |
| SII | 131.2 (48.4, 214.0)** | 94.0 (9.9, 178.0)* | 93.7 (5.4, 182.0)* | 57.7 (−37.9, 153.3) | 44.3 (−91.0, 179.6) | |
| SIRI | 0.51 (0.21, 0.81)** | 0.38 (0.08, 0.67)* | 0.36 (0.06, 0.67)* | 0.28 (−0.04, 0.60) | 0.10 (−0.15, 0.34) | |
| Blood Pressure Outcomes | | | | | | |
| Systolic BP, mmHg | 7.35 (3.63, 11.08)*** | 3.32 (−0.94, 7.58) | 2.54 (−2.17, 7.24) | 1.02 (−3.53, 5.57) | −0.13 (−6.41, 6.15) | |
| Diastolic BP, mmHg | 3.30 (0.11, 6.49)* | 4.02 (0.95, 7.09)* | 2.58 (−0.40, 5.57) | 0.50 (−2.61, 3.61) | 0.93 (−3.81, 5.67) | |

*Model 1: Unadjusted. Model 2: +age, sex, race/ethnicity, education, PIR. Model 3A: +smoking, waist circumference category. Model 3B: +smoking, continuous waist circumference. Model 4B: +physical activity, alcohol use, sleep duration. Significance: *p<0.05; **p<0.01; ***p<0.001.*

*Covariate selection rationale: Selected a priori based on clinical/epidemiological evidence linking covariates to both asthma and diabetes. No stepwise or statistical selection methods used. Continuous waist circumference used in primary model (Model 4B) to capture abdominal adiposity.*

**Appendix Table S4. Sensitivity analyses: associations of joint asthma–diabetes status across alternative model specifications (NHANES 2015–2020)**

| Model Specification | Asthma Only β (95% CI) | Diabetes Only β (95% CI) | Both β (95% CI) | Interaction P-value |
| --- | --- | --- | --- | --- |
| *Outcome: HbA1c (%) , Primary Sensitivity Analyses* | | | | |
| Model 4B (primary cont. waist circ.) | −0.01 (−0.10, 0.09) | 1.50 (1.29, 1.71)*** | 1.70 (1.38, 2.03)*** | 0.248 |
| Model 4A (BMI category instead of waist) | −0.00 (−0.09, 0.08) | 1.51 (1.29, 1.73)*** | 1.90 (1.36, 2.45)*** | 0.231 |
| Model 3B (no physical activity/sleep) | 0.01 (−0.04, 0.06) | 1.52 (1.37, 1.68)*** | 1.93 (1.55, 2.30)*** | 0.201 |
| Model 3A (BMI category, no PA/sleep) | 0.01 (−0.04, 0.06) | 1.53 (1.37, 1.68)*** | 1.96 (1.57, 2.34)*** | 0.215 |
| Model 2 (demographics only) | 0.03 (−0.02, 0.08) | 1.56 (1.41, 1.71)*** | 2.00 (1.61, 2.39)*** | 0.182 |
| Model 1 (unadjusted) | 0.04 (−0.01, 0.09) | 1.72 (1.59, 1.85)*** | 2.01 (1.61, 2.41)*** | 0.223 |
| *Outcome: Fasting Glucose (mg/dL) , Primary Sensitivity Analyses* | | | | |
| Model 4B (primary cont. waist circ.) | −0.04 (−2.26, 2.18) | 50.51 (39.70, 61.31)*** | 58.86 (45.49, 72.23)*** | 0.235 |
| Model 4A (BMI category instead of waist) | 0.16 (−2.11, 2.42) | 49.83 (39.28, 60.38)*** | 68.51 (48.21, 88.81)*** | 0.201 |
| Model 3B (no physical activity/sleep) | 0.39 (−1.09, 1.86) | 49.93 (43.80, 56.06)*** | 67.21 (53.91, 80.52)*** | 0.190 |
| Model 3A (BMI category, no PA/sleep) | 0.45 (−0.99, 1.90) | 50.13 (44.01, 56.25)*** | 68.29 (54.93, 81.66)*** | 0.185 |
| *Outcome: hs-CRP log-transformed — Sensitivity Analyses* | | | | |
| Model 4B (primary cont. waist circ.) | 0.29 (−0.10, 0.68) | 0.26 (0.07, 0.45)** | 0.74 (0.35, 1.12)*** | 0.649 |
| Model 4A (BMI category) | 0.30 (−0.03, 0.63) | 0.14 (−0.08, 0.36) | 0.33 (−0.19, 0.84) | 0.635 |
| Model 3B | 0.18 (−0.01, 0.37) | 0.12 (−0.00, 0.24) | 0.45 (0.08, 0.82)* | 0.584 |
| Model 2 | 0.28 (0.05, 0.51)* | 0.44 (0.32, 0.57)*** | 1.19 (0.87, 1.52)*** | 0.521 |

*Sensitivity analyses demonstrate robustness of primary findings across alternative adiposity measures (waist circumference vs. BMI category) and covariate sets. Results for HbA1c and fasting glucose remain consistent across specifications. hs-CRP associations are attenuated with continuous waist circumference adjustment, suggesting central adiposity as a partial mediator. Significance: *p<0.05; **p<0.01; ***p<0.001.*

**Appendix Table S5. Subgroup analyses by sex and central obesity status— joint asthma–diabetes status (NHANES 2015–2020)**

| Subgroup / Outcome | Asthma Only β (95% CI) | Diabetes Only β (95% CI) | Both β (95% CI) | P-interaction (Subgroup × Status) |
| --- | --- | --- | --- | --- |
| *Outcome: HbA1c (%), Subgroup: Sex* | | | | |
| Male (Model 4B) | −0.05 (−0.18, 0.08) | 1.46 (1.20, 1.72)*** | 1.61 (0.94, 2.27)*** | 0.610 |
| Female (Model 4B) | 0.03 (−0.11, 0.17) | 1.55 (1.27, 1.82)*** | 1.75 (1.34, 2.16)*** |  |
| *Outcome: HbA1c (%), Subgroup: Central Obesity* | | | | |
| Normal waist (Model 4B) | −0.04 (−0.22, 0.14) | 1.61 (1.24, 1.99)*** | 2.05 (0.91, 3.19)*** | 0.432 |
| High waist/central obesity (Model 4B) | 0.01 (−0.12, 0.14) | 1.44 (1.17, 1.70)*** | 1.64 (1.25, 2.03)*** |  |
| *Outcome: Fasting Glucose (mg/dL) , Subgroup: Sex* | | | | |
| Male (Model 4B) | −1.12 (−4.51, 2.27) | 51.0 (36.5, 65.5)*** | 68.0 (38.5, 97.5)*** | 0.738 |
| Female (Model 4B) | 0.95 (−2.20, 4.11) | 50.2 (35.4, 65.0)*** | 53.5 (35.3, 71.7)*** |  |
| *Outcome: Fasting Glucose (mg/dL) , Subgroup: Central Obesity* | | | | |
| Normal waist (Model 4B) | −2.14 (−7.02, 2.74) | 53.2 (36.7, 69.7)*** | 72.3 (39.5, 105.2)*** | 0.526 |
| High waist/central obesity (Model 4B) | 0.68 (−2.02, 3.38) | 49.3 (35.9, 62.8)*** | 56.0 (38.1, 73.9)*** |  |
| *Outcome: hs-CRP log-transformed , Subgroup: Sex* | | | | |
| Male (Model 4B) | 0.14 (−0.40, 0.68) | 0.20 (−0.09, 0.49) | 0.85 (0.22, 1.48)** | 0.515 |
| Female (Model 4B) | 0.41 (−0.13, 0.95) | 0.30 (0.04, 0.57)* | 0.65 (0.12, 1.18)* |  |
| *Outcome: SIRI , Subgroup: Sex* | | | | |
| Male (Model 4B) | 0.29 (0.07, 0.51)** | 0.19 (0.01, 0.37)* | 0.04 (−0.28, 0.36) | 0.714 |
| Female (Model 4B) | 0.38 (0.15, 0.61)*** | 0.24 (0.06, 0.43)** | 0.13 (−0.18, 0.44) |  |

*All models use Model 4B specification (adjusted for age, race/ethnicity, education, poverty–income ratio, smoking, continuous waist circumference, physical activity, alcohol use, sleep duration), restricted to each subgroup. Subgroup × Status interaction p-values test for effect modification. No significant interactions by sex or central obesity status were detected for any outcome, supporting generalizability of primary findings. Significance: *p<0.05; **p<0.01; ***p<0.001.*

These were all verified as correctly captured: N = 5,295 throughout, waist circumference as primary covariate, BH correction, RERI/AP/S computed, HDL-C P = 0.039 and SIRI P = 0.003 interactions, sub-additive framing, diabetes subtype limitation, self-report symmetry, clinical thresholds (158.8 mg/dL, 7.07%, 8.80 mg/L), Th2/Th1 distinction, corticosteroid discussion, visceral adiposity as shared driver, hypothesis-generating framing, all limitations, Mubanga/Chen cited, 42 references, Appendix Tables S1–S2.
